# Austin District Medical Society, Second Quarterly Meeting

**Published:** 1887-12

**Authors:** 


### AUSTIN DISTRICT MEDICAL SOCIETY.

SECOND REGULAR QUARTERLY MEETING, AUSTIN, TEXAS, DECEMBER 8th, 1887.

At 10 o'clock, A. M. Thursday December 8, pursuant to call by the Secretary of the Austin District Medical Society, the following physicians, resident in Austin, and in the adjacent counties, assembled in the hall of the Society.

President, Dr. T. D. Wooten............ ............Austin.
Vice-President, Dr. W. T. Richmond................Manor.
Secretary, Dr. T. J. Bennett.......... ...............Austin.
Dr. G. W. Christian........................... ...Burnet.
" L. C. Johnson..................................Giddings.
" Sam Cunningham...............................Elgin.
" G. W. Cain.......................................Elgin.
" R. Atkinson...............................San Marcos.
" W. M. Cunningham..........................Bastrop.
" L. D. Hill................................Webberville
" T. O. Maxwelle.............................Fiskville.
" F. R. Martin....................... ...........Kyle.
" R. S. Gregg.....................................Manor.
" M. K. Lott......................................Belton.
" G. W. Manney..................................Austin.
" B. E. Hadra......................................Austin.
" Florence E. Collins..............................Austin.
" A. N. Denton....................................Austin.
" F. E. Daniel......................................Austin.
" L. B. Johnson....................................Austin.
" J. W. Hamilton............................Webberville.
" R. H. Burleson............................Webberville.
" Q. C. Smith.......................................Austin.
" T. J. Tyner........................................Austin.
" J. W. McLaughlin...............................Austin.
" J. F. Dean.....................................Hornsby.
" F. T. Paine.......... ....................Comanche.
" W. A. Ellison.............................Manchaca.
" R. M. Swearingen...............................Austin.

Mr. Smith and Mr. Morris, students.

The President and Vice-President, being temporarely absent, Dr. W. A. Morris, the veteran physician of Austin, called the meeting to order, Secretary Bennett being at the desk.

Roll call showed a quorum present, and the Society proceeded to business.

Minutes of last meeting were read and approved.

A resolution by Dr. Hadra, read at last meeting proposing to change the By-laws with reference to appointing essayists alphabetically was brought up, and after discussion was put to vote and lost.

President Wooten having arrived, Dr. Morris gracefully yielded the gavel to him, and the regular order of business was taken up.

Applications for membership from the following gentlemen were read and were referred to the board of censors. The board consisted of Drs. A. N. Denton, W. A. Morris, R. S. Gregg, Sam Cunningham and F. E. Daniel. The board approved the applications, and on ballot the applicants were unanimously elected, to-wit :

Dr. T. J. Tyner....................................Austin.
" J. W. McLaughlin..................................Austin.
" R. Atkinson...................................San Marcos.
" W. M. Cunningham.................................Bastrop.
" L. C. Johnson..................................Giddings.
" W. S. Johnson..................................Giddings.
" G. W. Christian...................................Burnet.
" F. R. Martin.......................................Kyle.
" L. D. Hill.................................Webberville.
" M. K. Lott........................................Belton.
" J. F. Dean.......................................Hornsby.
" G. W. Cain.........................................Elgin.

Reading of essays was called for, but the hour of adjournment naving arrived, the Society took recess, till

2 O'CLOCK, P. M., when Dr. W. M. CUNNINGHAM, of Bastrop, read an account of

AN ABDOMINAL TUMOR, SUPPOSED TO BE OVARIAN, FOR WHICH LAPAROTOMY WAS PERFORMED, and which proved to be a very remarkable case of extra-uterine pregnancy in which the fœtus had become ossified.*

*) This paper is published in full in the present issue of the JOURNAL.

Dr. Daniel moved a vote of thanks to Dr. Cunningham, for his interesting and instructive paper; adopted.

The discussion on this paper was not as thorough as it might have been.

Dr. Morris, the oldest physician present, in a practice of fifty years, had never met a similar case:—he approved Dr. Cunningham's course in operating, and thought, as desperate as the operation apparently was, it afforded the only chance for the patient's life.

Dr. McLaughlin said the case was one of great interest, and we should profit by its teachings. It is very unfortunate that no autopsy was made, the indications being that the patient died of septic poisoning, and the doctor's course, in failing to use the bichloride solution and a drainage tube might, in the absence of positive indications to the contrary, be criticised. He inquired, what antiseptic was used, and in what strength? Dr. Cunningham replied, that he had used carbolized water of unknown strength, as the acid was not measured.

Dr. Daniel thought the collapse following a sudden and violent muscular effort of the patient rather pointed to hemorrhage, the sudden rupture of some vessel or ligature.

Dr. Wooten commended the operation under the c[illegible]umstances, but impressed the necessity of observing the most minute and careful details of antiseptic treatment in opening the abdomen; thinks in this case particularly, where the old abdominal pregnancy was associated with ascites, all the nice points of antisepsis should have been observed, and agreed with Dr. McLaughlin, that the omission of a drainage tube was unfortunate. Thomas, he stated, in suturing the abdominal wall, always sutures the peritoneum first, and he thinks, it would have been better, had it been done here. In all old and feeble subjects, like Dr. Cunningham's case, he much questioned the propriety of cold applications to the wound; they are too depressing; and an idiosyncrasy of this patient was that she was peculiarly susceptible to the effects of cold. Upon the whole it was a deserving paper, and he thought, taught a valuable lesson. Dr. Wooten then related a case, which occurred in his practice in early life, where a negro woman, ailing a long time with obscure abdominal trouble, passed fœtal bones per rectum, and got well.

Dr. Denton expressed some surprise at the extreme mobility of the tumor considering the numerous attachments.

Dr. Black, of Round Rock, sent in a paper which was read by Dr. Daniel. It was a report of a case of

GASTROTOMY FOR INCISED WOUND OF STOMACH,*

in which the contents of the stomach, including a number of large fish bones, were emptied into the free peritoneal cavity. The patient was present, his recovery, under the circumstance, being considered remarkable.

It will be observed in reading the paper, that the surgeons were so situated that they had to use what they could get,—and had not the approved appliances and dressings for abdominal surgery, at hand. The contents of the stomach, which had been spilled into the abdomen, were swobbed out with a cotton wet in common muddy creek water, held in an old tin pan, and the wound was dressed without the bichloride and other antiseptic† applications. The treatment in this case was criticised by those present, but the omission to treat the case in accordance with all the approved modern views was the force of circumstances.

The report of the case, just after Dr. Cunningham's, was a coincidence. There is an old lady, whose abdomen was opened, and who died, it was supposed, because drainage and strict antisepsis were not used. Here is a young man whose stomach is cut into, and emptied into the abdominal cavity, who lies twenty-four hours in agony, and without medical attention, and whose case is also treated without the approved bichloride and drainage—and not a drop of water used to cleanse the cavity other than that afforded from cotton wetted with creek water; and yet he recovers without a bad symptom! What estimate is to be put on the use of antiseptics?

The discussion of this paper was brief.

Dr. Hadra called attention to the very extraordinary success of Tait and Bantock in abdominal surgery notwithstanding their well known disregard of *all* antiseptic precautions; they using only "tap water," and enforcing the most absolute cleanliness. [But, in Dr. Black's case, it would seem that

---

*) This paper appears in full in this issue of the Journal.
†) Except iodoform.

it would be impossible to thoroughly cleanse the abdominal cavity by the means employed ; how account for the recovery of the patient?] Dr. Hadra also criticised the mode of incision in this case, obliquely: insisting that it should, in such cases, be made in the median line, and cited authority.

Dr. Wooten inquired if the peritoneal suture were included in the suture of the wound ? and pointed out the advantage of separate sutures for the peritoneal coat.

Dr. Denton and Dr. Christian also discussed this paper, and on motion of Dr. Christian, a vote of thanks was tendered Dr. Black for his interesting paper.

Dr. L. D. Hill, of Webberville, read a paper on Septic Poisoning,* in which he reviewed the literature of the subject, giving the views af a number of leading authorities, stating his own, and giving his experience with wounds in the confederate hospitals during the war.

Dr. Bennett moved that the paper be accepted, and Dr. Hill thanked for his interest and labors in presenting it; adopted.

Discussion in this paper was postponed because Dr. Morris had one on Typhoid Fever; and as the subject of germ causation and infection was embraced in each, it was proposed to discuss them jointly.

Dr. W. A. Morris made many apologies for his paper, saying that he had not given that care to its preparation which the importance of the subject demanded, but had written it at request of members, and to evince his interest in the effort to make the society and its meetings a success.

Dr. Wooten said that anything from the pen of Dr. Morris, the veteran practitioner of Austin, who had given fifty years to the study and practice of medicine, would surely prove interesting and valuable, and it was urged that the doctor read his paper. He did so; it was entitled,

TYPHOID FEVER AND ITS TREATMENT.†

[The discussion on this and the preceding paper took a wide range, and was participated in by most of the members. We regret that we are unable to give here, even a synopsis of either the pa-

*) This paper will appear in next issue of the Journal.
†) This paper will appear very soon.

pers, or the discussion, but will endeavor to do so when the papers appear in the JOURNAL. ED.]

DR. RICHMOND moved a vote of thanks to Dr. Morris; carried.

DR. DANIEL called attention to the fact that the venerable Dr. Paine, of Comanche, who had been invited to meet with the society for the purpose of expounding and elucidating his peculiar views on the subject of electricity in nervous diseases, had entered the room, and suggested that an hour be appointed to hear him. It was therefore agreed that his lecture take place at the evening meeting, at 8 oclock, and on motion the society adjourned till 7-30 p. m.

7-30 P. M. Business resumed, Dr. Wooten in the chair.

DR. T. J. TYNER'S paper came next on the programme, and was called for.

DR. TYNER read a paper entitled

EYE DISEASES AND OTHER REFLEXES DUE TO ERRORS OF REFRACTION.‡

DR. Q. C. SMITH called attention to the importance of a study of the eyes and their condition, in school children, and thought it was the duty of the medical profession to point out to parents and teachers the nature of these defects, their frequent existence unsuspected, and instruct them in the mode and manner of their relief, (or prevention).

DR. TYNER stated that he had often volunteered such information, but got no satisfaction; that, as a rule, no action was had. He stated that children are sometimes punished for indolence, when their only sin was *myopia* or *hypermetropia.*

DR. LOTT concurred in the remarks of Drs. S. and T., and stated that he had discovered, in his oldest daughter, the existence of a visual defect which hindered her at school, and that he had suitable glasses adjusted, but the teacher made her take them off, saying that "glasses were for old people, and made children look ridiculous." But he insisted that she should wear them, and the myopia and the *granulated lids* which had occurred as a consequence, were both cured.

DR. MCLAUGHLIN said, in view of the great importance of the subject to the profession and the laity, as well as the value of the paper, it should be published; and moved that it be published in

‡) Published in this number of the JOURNAL by vote.

DANIEL'S TEXAS MEDICAL JOURNAL in order that it might be extensively read, especially by Texas physicians.

DR. BENNETT stated that Dr. Daniel had kindly published all announcements and other matters of interest and importance concerning this society, and had also placed the JOURNAL at their further service; and he moved, therefore, that the society return thanks to Dr. Daniel, and accept the use of the JOURNAL, making it the OFFICIAL ORGAN of the society. Seconded by Dr. Wooten, and unanimously adopted.

It was also moved and adopted, that a committee of publication be appointed, consisting of three, (of whom the editor of the JOURNAL should be one) and to whom all papers read at the meetings of the Austin District Medical Society should be referred; and that they select such papers for publication in the JOURNAL as, in their judgement, have merit to justify it, or contain matter of sufficient interest and importance to the profession; adopted. The chair asked Dr. Daniel to indicate a preference for the appointment of his two colleagues, but Dr. Daniel begged to be excused,saying that he was happy in the belief, nay, assurance, that he possessed the confidence and esteem, as well as the good will of the entire profession of Austin, and that that feelling was mutual; that the appointment of any two members would be agreeable to him, while to indicate a preference would perhaps look invidious; and he really could not, where all were so capable and so acceptable, make a choice. Thereupon, the chair appointed Dr. J. W. McLaughlin and Dr, T. J. Bennett to act with Dr. Daniel as the publishing committee of the society.

DR. MENGER, of San Antonio, had sent up a paper which Dr. Daniel rose to read, it being entered next on the programme; but as it was the hour assigned to Dr. Paine, and it was objected that no paper from an absentee should be read while there were any present still to be heard, Dr. Daniel, of course, gave way to Dr. Paine, who gave the soaiety a clear and most interesting exposition of his theory as to electrical anæsthesia in certain diseases, and his method of diagnosis based thereon.*

---

*) As Dr. Paine's views have been published in the JOURNAL heretofore, and especially as we have not room now, further mention is postponed till we can publish his paper in full.

Dr. Hadra moved that a vote of thanks be returned to Dr. Paine. He was an old gentleman, and an old physician, and had come four hundred miles in order that he might do us the honor to be present at our meetiug, and had treated the sociely to an able and interesting lecture. The motion was seconded and unanimously adopted.

Dr. Q. C. Smith, by invitation, exhibited some instruments of his own invention and manufacture, and demonstrated their use in the treatment of strangulated hernia.

Essayists appointed for next meeting were Drs. S. Cunningham and L. A. Cocke, with Dr. Davidson (of Manchacha) and D. Denton as alternates.

The time and place of next meeting was fixed at Austin, the early part of March, 1888; notice of the exact day to be given by the secretary in due time.

The meeting adjourned for three months, and the visiting members departed, expressing themselves well pleased and much edified by their attendance.

[The association is in a very fair way to acquire strength and importance, and to become a feature in the grand scheme of organizing the regular profession of the State, an object to which this Journal it devoting its best energies.—Ed.)

